# Characterization of a novel + 70 Da modification in rhGM-CSF expressed in *E. coli* using chemical assays in combination with mass spectrometry

**DOI:** 10.1007/s00726-021-03004-9

**Published:** 2021-08-28

**Authors:** Magdalena Widgren Sandberg, Jakob Bunkenborg, Stine Thyssen, Martin Villadsen, Thomas Kofoed

**Affiliations:** 1 Universitätsklinikum Hamburg-Eppendorf (UKE), Hamburg, Germany; 2 Alphalyse A/S, Odense, Denmark

**Keywords:** Post-translation modification, Recombinant protein production, Crotonaldehyde, *E. coli*, Granulocyte-macrophage colony-stimulating factor

## Abstract

Granulocyte-macrophage colony-stimulating factor (GM-CSF) is a cytokine and a white blood cell growth factor that has found usage as a therapeutic protein. During analysis of different fermentation batches of GM-CSF recombinantly expressed in *E. coli*, a covalent modification was identified on the protein by intact mass spectrometry. The modification gave a mass shift of + 70 Da and peptide mapping analysis demonstrated that it located to the protein N-terminus and lysine side chains. The chemical composition of C_4_H_6_O was found to be the best candidate by peptide fragmentation using tandem mass spectrometry. The modification likely contains a carbonyl group, since the mass of the modification increased by 2 Da by reduction with borane pyridine complex and it reacted with 2,4-dinitrophenylhydrazine. On the basis of chemical and tandem mass spectrometry fragmentation behavior, the modification could be attributed to crotonaldehyde, a reactive compound formed during lipid peroxidation. A low recorded oxygen pressure in the reactor during protein expression could be linked to the formation of this compound. This study shows the importance of maintaining full control over all reaction parameters during recombinant protein production.

**Supplementary Information:**

The online version contains supplementary material available at 10.1007/s00726-021-03004-9.

## Introduction

Since the first human insulin was produced through recombinant DNA technology using *E. coli* as expression host, the technique of recombinant protein expression has come to revolutionize the biomedical field (Johnson 1983). The efficient production of proteins in host cells requires highly optimized reaction conditions to obtain high yields while avoiding unwanted protein variants. Different protein variants, or proteoforms, can for example derive from sequence variants, truncations, post-translational modifications (PTMs), or incorporation of non-canonical amino acids (Farr’ and Kogoma 1991; Rehder et al. 2008; Valdez-Cruz et al. 2011; Wang et al. 2011). These proteoforms may affect the drug’s biological activity, pharmacokinetics, pharmacodynamics, or immunogenicity, and thereby also affect the drug safety.

Granulocyte-macrophage colony-stimulating factor (GM-CSF, https://www.uniprot.org/uniprot/P04141) is a cytokine and a white blood cell growth factor which is used in the lungs to regulate surfactant homeostasis and the lungs’ host defense (Francisco-Cruz et al. 2014). Disruption of the surfactant homeostasis by GM-CSF autoantibodies leads to a condition called autoimmune Pulmonary Alveolar Proteinosis and can be treated by administration of external GM-CSF to the patient (Tazawa et al. 2010). The mature form of human GM-CSF is a protein containing 127 amino acids and four cysteine residues forming two disulfide linkages (Schwanke et al. 2009). It is a glycoprotein with two N-glycosylation sites and several O-glycosylation sites.

Since the molecular cloning and expression of recombinant human GM-CSF (rhGM-CSF) in 1985, biologically active forms of the protein have been expressed in multiple systems including *E.coli*, yeast, plant, and mammalian cells (Wong et al. 1985; Forno et al. 2004; Zhou et al. 2006). *E. coli* has been widely used due to its ability to grow rapidly at high density and on inexpensive substrates (Rosano and Ceccarelli 2014). Furthermore, *E. coli* lacks a system for addition of PTMs like glycosylation which limits the number of possible proteoforms (Sahdev et al. 2007). GM-CSF is expressed in *E. coli* as the active sequence, without the signal peptide, and with a translation initiating methionine residue on the protein N-terminal (Thomson et al. 2012). The initiating methionine is then removed by proteases. The rapid cell growth and high possible recombinant protein yield of *E. coli* implies that the system demands a high supply of nutrients. Oxygen has a limited solubility in the medium and requires proper mixing in the fermentor to keep up the oxygen supply to the cells (Konz et al. 1998). A lack of oxygen supply to the cells, hypoxia, has been shown to activate oxidative cell responses leading to excessive production of reactive oxygen species and subsequent lipid peroxidation (Joanny et al. 2001; Clanton 2007). Many of these lipid peroxidation products are susceptible to attack by nucleophilic protein side chains like cysteine, histidine, arginine, and lysine residues. The most important products of lipid peroxidation giving rise to protein modification are reactive aldehydic intermediates like ketoaldehydes, 2-alkenals, and 4-hydroxy-2-alkenals. These may pose a possible source to the formation of unwanted protein PTMs (Ichihashi et al. 2001; Domingues et al. 2013; Afonso et al. 2018).

In this work, we have identified and characterized a novel modification in recombinant GM-CSF process samples expressed in a strain of *E. coli*. The modification was identified in an early stage, unoptimized development fermentation in which the fermentation conditions were poorly controlled with respect to aeration and nutrient feeding. By analyzing the molecular weight of the intact protein and by peptide mapping with LC–MS, the adduct was found to add a mass of 70 Da to the protein N-terminal and lysine side chains, and by peptide fragmentation, the elemental composition could be determined. Various chemical assays were used to probe the chemical composition of the adduct demonstrating that it contains a carbonyl group.

## Materials and methods

### Chemicals

Urea, sodium phosphate dibasic dihydrate, sodium phosphate monobasic dihydrate, 1,4-Dithiothreitol (DTT), iodoacetamide, N-ethylmaleimide, borane pyridine complex, 4-Vinylpyridine, triethylammonium bicarbonate buffer (TEAB), 3-buten-2-one (MVK), and formic acid (FA) were all purchased from Sigma-Aldrich. Trifluoroacetic acid (TFA) (Acros Organics), acetic acid (Chemsolute), DNPH (Tokyo Chemical Industry Co., Ltd.), dimethyl sulfoxide (DMSO) (Thermo Scientific), and acetonitrile (ACN) (Chemsolute).

### Protein digestion

The methionylated GM-CSF development sample was denatured and alkylated in 6 M urea, in 50 mM phosphate buffer with pH 7, and 5 mM iodoacetamide for 1 h at 30 °C protected from light. The sample was diluted in phosphate buffer to 0.8 M urea before digestion over night with LysC (Lysyl EndopeptidaseR; FUJIFILM Wako Pure Chemical Corporation) and GluC (sequencing grade; Promega, Madison, WI), enzyme-to-protein ratios 1:10 and 1:25, respectively at 30 °C. The digestion was stopped with 1% TFA.

### Carbonyl reduction using borane pyridine complex

The methionylated GM-CSF development sample was denatured and reduced in 6 M urea, in 50 mM phosphate buffer with pH 6, and 100 mM borane pyridine complex over night at room temperature. The buffer was exchanged on Vivaspin 5 kDa molecular weight cut-off (MWCO) filters (Sartorius) to 6 M urea in phosphate buffer and the volume was reduced to 25 µL. The sample was then reduced in 5 mM DTT for 1 h at 30 °C followed by alkylation with 10 mM 4-vinylpyridine for 45 min at room temperature. The sample was then digested with LysC, 1:10 enzyme-to-protein ratio, for 2 h at 30 °C, followed by dilution in phosphate buffer to a urea concentration of 0.8 M, and then digested with GluC, 1:25 enzyme-to-protein ratio, overnight at 30 °C. The digestion was terminated with 1% TFA.

### Aldehyde/ketone DNPH derivatization

A solution of 100 mM DNPH and 0.5% TFA in DMSO was prepared. 7.5 µg digested methionylated GM-CSF development sample, according to the protocol for *Protein digestion* described above, was evaporated by vacuum centrifugation. 25 µL of the DNPH solution was added to the protein and the solution was left in a shaker at room temperature overnight.

### MVK and crotonaldehyde derivatization

Five µL native recombinant GM-CSF (2.23 mg/mL) with low degree of + 70 Da modification was diluted in 40 µL 100 mM TEAB, pH 8.5. 5 µL MVK or crotonaldehyde in ultra-high-quality (UHQ) water with the concentrations 10 µM, 100 µM, 1 mM, 10 mM, and 100 mM was added to achieve final concentrations of 1 µM, 10 µM, 100 µm, 1 mM, and 10 mM, and the samples were incubated for 24 h at 37 °C. The sample buffers were changed to 6 M urea in 50 mM NaP, pH 7 using Vivaspin 5 kDa MWCO filters (Sartorius) before protein digestion according to the *Protein digestion* protocol described above.

### Peptide SPE by HLB elution plate

MVK and crotonaldehyde derivatized digests were cleaned up prior to *RP-LC-ESI-TripleTOF-MS* analysis on HLB µElution plate (Oasis). The filters were activated with 100% MeOH followed by equilibration with UHQ water before adding 5 µg sample diluted 1:1 in 4% phosphoric acid. The bound sample was washed with 5% MeOH followed by elution in 100% MeOH. The peptides were dried by vacuum centrifugation before being dissolved in 20 µL 0.1% formic acid (FA) in UHQ water.

### Intact protein analysis by RP-LC-ESI-QTOF-MS

LC–MS analyses of the intact proteins were performed on an Agilent 1290 Infinity II system with a variable wavelength detector coupled to a Bruker Maxis Impact mass spectrometer. 10 µg GM-CSF in 40 µL 0.1% FA was loaded on an ACQUITY UPLC Protein BEH C4 Column, 300 Å, 1.7 µm, 2.1 mm × 150 mm (Waters), operated at 60 °C column oven temperature. Elution was performed at a flow rate of 0.2 mL/min with solvent A (0.1% TFA in UHQ water) and solvent B (0.1% TFA in 90% ACN). A linear gradient of 36–56% solvent B was applied for 30 min followed by column washing and reconditioning. MS data were recorded in the range 500–3000 m/z. The data were deconvoluted in DataAnalysis (Bruker) using the MaxEnt algorithm.

### Peptide mapping by RP-LC-ESI-QTOF-MS

Peptide mapping of the methionylated GM-CSF development sample was performed using an Exion system coupled to an SCIEX x500b mass spectrometer. The protein digest was loaded directly on an Xselect CSH C18 XP column, 130 Å, 2.5 µm, 2.1 × 150 mm (Waters) at 60 °C column oven temperature. 1.2 µg digest was loaded of the non-treated and the pyridine borane complex treated GM-CSF and 5 µg of the DNPH treated digest. Elution was performed at a flow rate of 0.2 mL/min with solvent A (0.1% FA in UHQ water) and solvent B (0.1% FA in ACN). The sample was washed for 6 min with 1% solvent B, letting the flow-through go to waste, before applying a linear gradient of 1–50% solvent B for 26 min, while letting the sample enter the mass spectrometer. This was followed by column washing and reconditioning. Mass spectrometry analysis was performed in positive polarity mode. MS data were recorded in the range 300–1800 m/z with an accumulation time of 0.5 s and a total cycle time of 1.2 s. MS/MS acquisition was performed in information-dependent mode (IDA) on charge states 2–5 exceeding 200 cps on a maximum of 13 candidate ions and excluding former candidate ions for 5 s after 2 occurrences, MS/MS scan range 130–2000 m/z.

### Peptide mapping by RP-LC-ESI-TripleTOF-MS

Peptide mapping of the MVK and crotonaldehyde derivatized native recombinant GM-CSF were performed using an Eksigent system coupled to a SCIEX TripleTOF 6600 mass spectrometer. 1 µg digest in 0.1% FA in UHQ water was loaded on a nanoEase M/Z CSH130 1.7 µm 300 µm × 150 mm column (Waters) at 60 °C column oven temperature. Elution was performed at a flow rate of 5 µL/min with solvent A (0.1% FA in UHQ water) and solvent B (0.1% FA in ACN). The column was equilibrated for 2 min at 5% solvent B before applying a linear gradient of 5–27% solvent B over 23 min, followed by column washing and reconditioning. Mass spectrometry analysis was performed in positive polarity mode. MS data were recorded in the range 300–1700 m/z with an accumulation time of 0.2 s and a total cycle time of 1.3 s. MS/MS acquisition was performed in information-dependent mode (IDA) on charge states 2–5 exceeding 100 cps on a maximum of 25 candidate ions and excluding former candidate ions for 3 s after 1 occurrence, MS/MS scan range 130–2000 m/z.

### Processing of peptide mapping data

.wiff2 files (from ESI-QTOF-MS) and .wiff files (from ESI-TripleTOF-MS) were converted to .mgf files using ProteoWizard’s MS convert program (version 3.0.18204 64-bit). The Mascot probability-based search engine was then used to search .mgf files against a protein database containing 10 sequence variants of the GM-CSF protein. Variable modifications included in the search were carbamidomethyl on cysteine residues, butyryl on peptide N-terminal, lysine, histidine and cysteine residues, glutamine to pyroglutamate and methionine oxidation. The .wiff2 and .wiff files were then analyzed quantitatively in Skyline (version 19.1.0.193) using a library created from the Mascot search results.

The theoretical elemental compositions and correlated isotopic masses presented in Table 1 were calculated using the “Molecular Weight Calculator” provided by the Pacific Northwest National Laboratory website (https://omics.pnl.gov/software/molecular-weight-calculator).

**Table 1:** Theoretical elemental compositions with a ± 0.05 Da mass deviation from the identified + 70 Da modification

Elemental composition	Theoretical mass [Da]	Mass deviation [ppm]	Sources
C_2_NO_2_	69.992904	706.18	
CN_3_O	70.004137	545.81	
C_3_H_2_O_2_	70.0054792	526.65	Pyruvic acid (Liu et al. 2011)
N_5_	70.01537	385.43	
C_2_H_2_N_2_O	70.0167122	366.27	
CH_2_N_4_	70.0279452	205.90	
C_3_H_4_NO	70.0292874	186.73	
C_2_H_4_N_3_	70.0405204	26.36	
C_4_H_6_O	**70.0418626**	**7.20**	Crotonaldehyde (Ichihashi et al. 2001), methyl vinyl ketone (von Stedingk et al. 2010), butyryl-CoA (Chen et al. 2007)
C_3_H_6_N_2_	70.0530956	153.18	
C_4_H_8_N	70.0656708	332.71	
C_5_H_10_	70.078246	512.25	Pentanal (Afonso et al. 2018)

The composition’s monoisotopic mass and its deviation from the experimental value of the + 70 Da modification, 70.042 Da, is given

All protein concentrations were measured on intact protein by amino acid analysis.

## Results

### Discovery of a + 70 Da modification

**Fig. 1:**
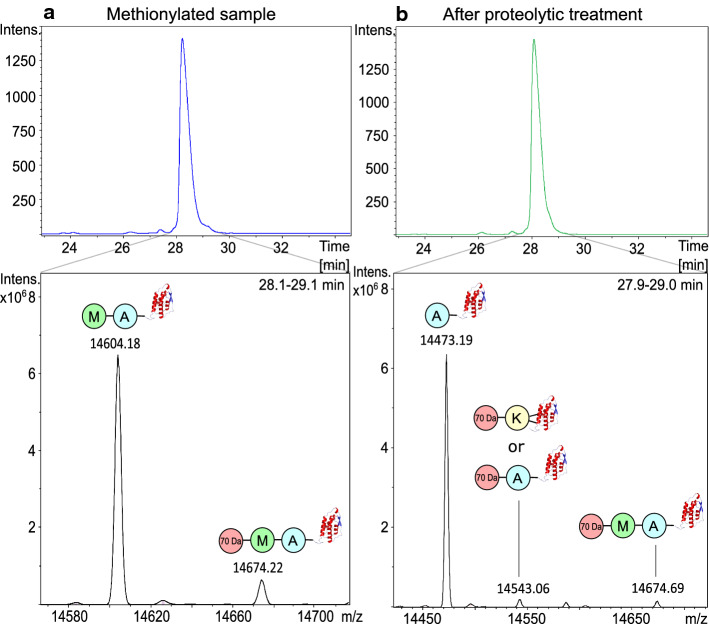
Two development samples from different steps in the production of recombinant human GM-CSF were analyzed as intact proteins by RP-LC coupled to UV (upper picture) and ESI–MS. The MS data from the main UV peak were deconvoluted to obtain the most abundant proteoforms (lower picture). The intact proteins elute at 28 min. **A** A methionylated sample, before addition of proteases cleaving excessive N-terminal methionine, contained two species: the methionylated GM-CSF and the same species with an additional mass of 70 Da. **B** A sample which had been through all processing steps contained mainly the native protein (with an N-terminal alanine residue) but also lower amounts of the native protein + 70 Da and the methionylated protein + 70 Da. The location of the modification cannot be determined using intact mass analysis

A set of protein production samples underwent analysis by mass spectrometry to characterize the product. A development sample of GM-CSF recombinantly expressed in *E. coli,* was analyzed during quality control by reversed phase coupled to UV-LC–MS in an intact, non-reduced state. The sample, which had been through all processing steps, appeared as a single peak by UV detection, as illustrated in Fig. 1B, upper picture. However, several species were detected after deconvolution of the charge state envelope of the main peak, as illustrated in Fig. 1B, lower picture. The peak contained the expected major proteoform with the mass 14,473.57 Da, corresponding to the molecular mass of native GM-CSF (theoretical mass 14,473.36 Da). The peak additionally contained one less abundant proteoform which was 70.40 Da heavier than the main proteoform, average mass 14,543.76 Da. This molecular form could not be attributed to any known proteoform. Another lower abundant proteoform with the mass 14,674.85 Da could be identified, matching the mass of the protein before proteolytical cleavage of the N-terminal methionine plus 70.30 Da. Analysis of an early process sample from the same batch, which had not been subjected to proteolytic cleavage of the N-terminal methionine, confirmed that this sample also contained several proteoforms from which two were in high abundance, Fig. 1A. The most abundant form had an average mass of 14,604.73 Da, corresponding to the mass of GM-CSF still containing the N-terminal methionine (theoretical mass 14,604.55 Da). The second most abundant proteoform had the average mass 14,674.86 Da, which is 70.31 Da heavier than the N-terminally methionylated GM-CSF. The fact that the methionine in the final product had been fully cleaved off except from a part of the + 70 Da modified peptide suggests that the modification interferes with the efficient removal of the N-terminal methionine. A commercially available batch of GM-CSF (Cat # Y0000251, EDQM) was analyzed for reference but no + 70 Da modification was identified in the sample, see Appendix Fig. 6.

To further study the proteoforms with the mass increase of 70 Da and to locate the adduct in the protein sequence, the methionylated development sample was characterized by peptide mapping using specific enzymes LysC and GluC. Trypsin, which is more commonly used for peptide mapping, was not used since the protein contains an arginine at amino acid site 4 (site 5 before N-terminal methionine processing) and we wanted to receive full sequence information on the protein N-terminal. The peptide mapping data acquired by LC–MS/MS was searched in Mascot followed by relative quantification in Skyline using a library generated from the Mascot search hits. The modification was found to be mainly located on the protein N-terminal methionine and to a smaller extent on lysine residues and on the non-methionylated N-terminal, see Appendix Fig. 7. The modification also seemed to be stable in the sense that it did not show any loss of OH or H_2_O ions upon peptide ionization in the ESI source.

### Characterization of a novel + 70 Da modification

**Fig. 2:**
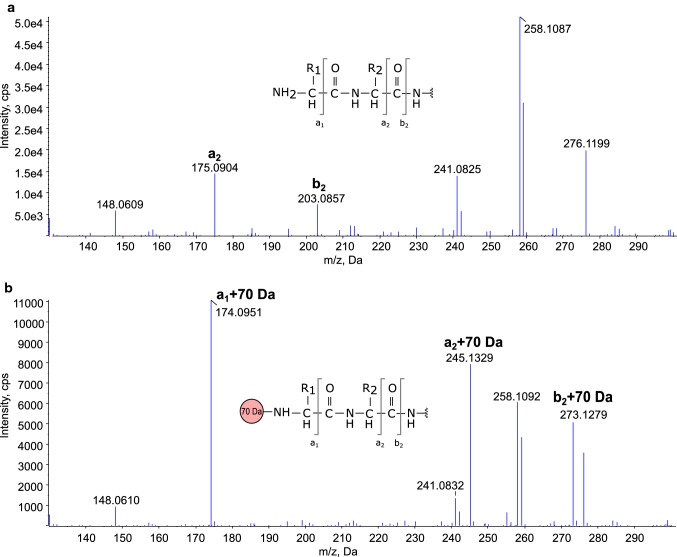
MS/MS spectra from low m/z ions after fragmentation of the N-terminal peptide MAPARSPSPSTQPWEHVNAIQE in the methionylated development sample of GM-CSF. The spectra from the 3 + charged precursor ion of **A** the non-modified peptide and **B** the + 70 Da modified peptide are shown. The modification mass was calculated by taking the m/z difference between the daughter ions from the two peptides for ions *a*_1_, *a*_2_, and *b*_2_

To identify the chemical composition of the + 70 Da modification that was found in the methionylated development sample of recombinant GM-CSF, fragment ions from the modified and non-modified N-terminal peptides were used to calculate the mass of the modification. By calculating the difference between the fragment ion m/z values from the two peptides, fragment ions *a*_1_, *a*_2_, and *b*_2_ were used to determine the mass of the adduct with high accuracy, giving masses 70.0422 Da, 70.0425 Da, and 70.0424 Da, respectively, see Fig. 2. The theoretical value of the a_1_ fragment from the non-modified peptide was used, since only ions above 130 m/z were recorded. The mean of the calculated masses, 70.0424 m/z, was then matched against several theoretical adduct masses, see Table 1.

**Fig. 3:**
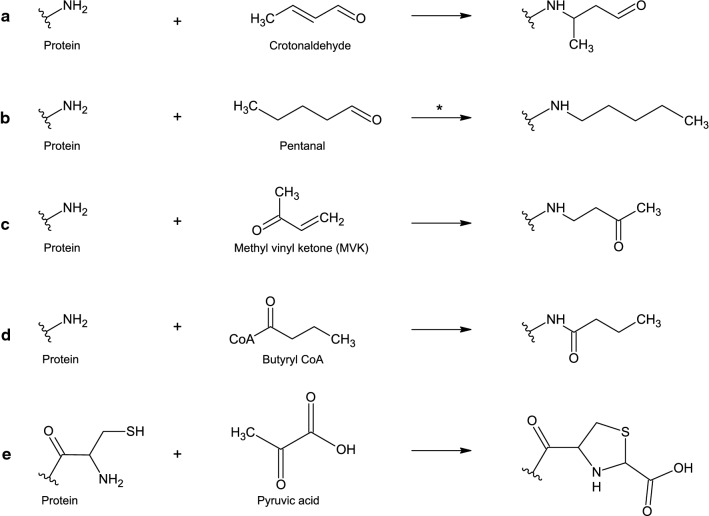
Protein primary amine modifications of + 70 Da reported in literature** A** (Ichihashi et al. 2001), **B** (Afonso et al. 2018), the depicted reaction product is the reduced Schiff base, **C** (von Stedingk et al. 2010), **D** (Chen et al. 2007), **E** (Liu et al. 2011)

In the literature, there are a number of protein adducts described with a mass of 70 Da. One such derivative is from reaction with crotonaldehyde, a 2-alkenal that appears as a by-product from oxidative reaction pathways in biological systems (Farr’ and Kogoma 1991; Esterbauer et al. 1991; Ichihashi et al. 2001). Crotonaldehyde has a high reactivity towards lysine side chains, leading to addition of a butanal group (C_4_H_6_O) by Michael addition (Fig. 3A). Another aldehyde that has been found to form adducts with the lysine side chain is pentanal, in this case through a Schiff’s base reaction resulting in addition of a C_5_H_10_ group (Fig. 3B) (Afonso et al. 2018). Ketones has also been found to react with protein side chains. Adducts of ethyl vinyl ketone (EVK) and methyl vinyl ketone (MVK) on N-terminal valine from Michael addition has been identified in human blood samples, the later one resulting in addition of the chemical composition C_4_H_6_O (Fig. 3C) (von Stedingk et al. 2010; Carlsson et al. 2015). In another study, it was shown that in a similar way as lysines can be acetylated by acetyltransferases using acetyl-CoA, lysines can be butyrylated through the metabolic intermediate structure butyryl-CoA (Fig. 3D) (Chen et al. 2007; Xu et al. 2018). In further another study, a + 70 Da modification was identified on the protein N-terminal cysteine of a recombinant protein expressed in *E. coli* (Liu et al. 2011). The adduct was hypothesized to have the chemical composition of pyruvate and be the result of reaction with pyruvic acid (Fig. 3E).

Both the mass of the pyruvic acid derivative and that of the pentanal derivative reported in the literature deviate from the observed mass by around 500 ppm, see Table 1. Moreover, pyruvic acid was derivatized with GM-CSF in a separate experiment and analyzed by peptide mapping and LC–MS/MS. The modification was found not to possess similar properties as the + 70 Da modification identified in the GM-CSF process samples, as described in Online Resource 1. The elemental composition C_4_H_6_O was the only composition that gave a mass deviation within the expected accuracy of the instrument, < 10 ppm, with a mass deviation of 7.20 ppm. It was therefore hypothesized that this was the elemental composition of the observed adduct. C_4_H_6_O matches the elemental composition of the isomeric adducts from crotonaldehyde, MVK, and butyryl-CoA described in the literature, see Fig. 3A–D.

**Fig. 4:**
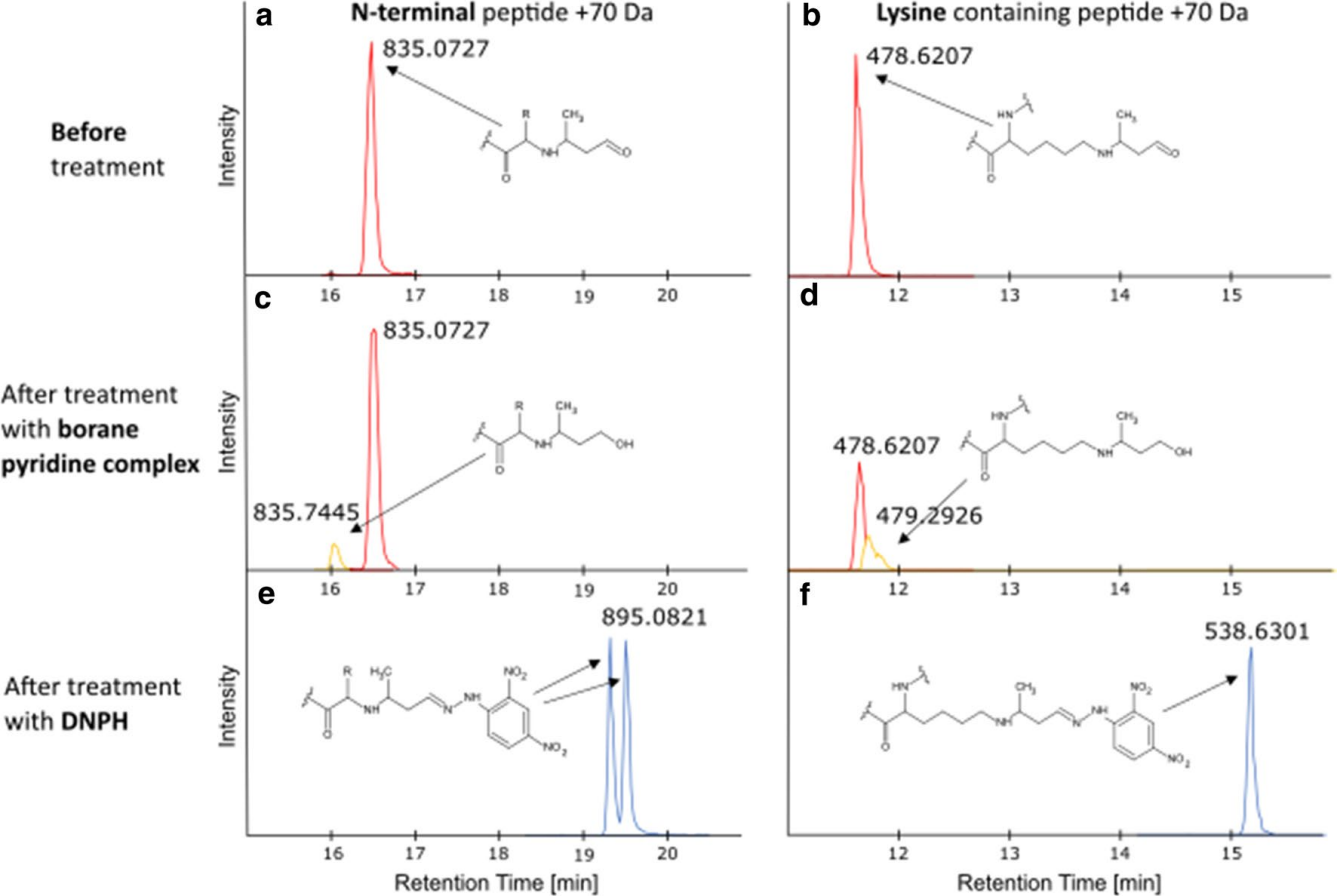
XICs of peptide precursor ions containing the endogenous + 70 Da modification on the protein N-terminal, peptide M[+ 70]APARSPSPSTQPWEHVNAIQE, or on lysine, peptide LYK[+ 70]QGLRGSLTK. **A** N-terminal peptide with no chemical treatment, **B** lysine containing peptide with no chemical treatment,** C** N-terminal peptide reduced with borane pyridine complex,** D** lysine containing peptide reduced with borane pyridine complex,** E** N-terminal peptide reacted with DNPH, and **F** lysine containing peptide reacted with DNPH. The sample data were acquired by LC–MS/MS. m/z values are shown for the precursor ions with charge state 3

To determine the chemical structure, and thereby the source, of the adduct, two chemical reactions were performed on the methionylated development sample. The reaction products from crotonaldehyde and MVK both contain a carbonyl group which should be reducible by a reducing agent, such as borane pyridine complex (Barnes et al. 1958). To reduce an adduct like butyryl where the carbonyl is involved in an amide bond, borane pyridine complex would not be a strong enough reducing agent. To test for the presence of a reducible double bond in the adduct, the intact methionylated development sample was therefore reduced with 50 mM borane pyridine complex for 1 h. The sample was digested with LysC and GluC followed by data acquisition with LC–MS/MS. The data showed that upon reduction of the protein a peptide peak started to appear from incorporation of two hydrogen atoms in the + 70 Da, thus a mass change to 72 Da. This was observed for both the modified protein N-terminal peptide and for modified lysine containing peptides, Fig. 4A–D. Butyryl-CoA could thereby be excluded as source to the + 70 Da modification. To further establish that the reducible double bond was located in a carbonyl group, the digested methionylated development sample was incubated with 100 mM 2,4-dinitrophenylhydrazine (DNPH), a classic reagent for carbonyls (Allen 1930). After incubation overnight, the + 70 Da adduct was completely converted to + 250 Da, corresponding to the expected mass after reaction with DNPH (Fig. 4E and F). Two peaks with different retention times were recorded from the DNPH derivatized N-terminal peptide. The two peaks can be explained by isomerization around the double bond between the carbonyl carbon in the + 70 Da modification and the nitrogen in DNPH. Peak splitting of diasteromers on reversed-phase HPLC has for example been observed in methionine oxidation products and occurs when the peptide’s secondary structure is affected (Lao et al. 2015). This isomerization was not observed for the DNPH derivatized lysine peptides which could be attributed to lack of peak separation or lack of effect on the peptides’ secondary structure. The fact that the + 70 Da modification could be derivatized with DNPH confirms the presence of a carbonyl moiety in the modification. Both the reaction product from crotonaldehyde and MVK contains a carbonyl moiety.

### Reconstruction of the modification

Two peptides with identical amino acid sequence and modifications should in theory interact similarly on a chromatographic column and provide similar fragments in the MS/MS collision cell. It was therefore examined if the + 70 Da modification could be reproduced in vitro using commercially available MVK and crotonaldehyde. The reactions were tested on a native recombinant GM-CSF which contained very low levels of + 70 Da modified protein and in which the N-terminal methionine had been proteolytically removed. The protein was reacted for 24 h at room temperature with different concentrations of MVK or crotonaldehyde followed by sample cleanup using molecular weight cut-off filters and analysis by peptide mapping and LC–MS/MS. The properties of the peptides containing the artificially produced modifications were compared with those of the peptides from the methionylated development sample containing the endogenous modification.

**Fig. 5:**
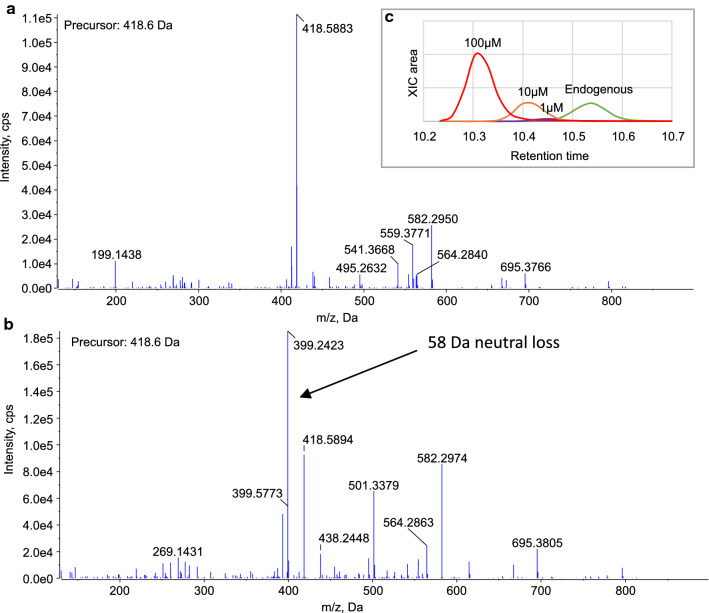
MS/MS fragmentation spectra from the 3 + charged precursor ions of peptide Q[+ 17]GLRGSLTK[+ 70]LK with **A** the endogenous + 70 Da modification, identified in the methionylated development sample of GM-CSF, and **B** the MVK reaction product. **c** shows the XICs from the same precusor ion overlaid from four samples: the methionylated development sample with the endougenous + 70 Da modification (green), the native recombinant GM-CSF reacted with 1 µM MVK (blue), 10 µM MVK (yellow), and 100 µM MVK (red)

Upon incubating the native recombinant GM-CSF with increasing levels of MVK, increasing levels of + 70 Da modified lysine could be observed, see Fig. 5C. The retention times of the artificially modified peptides were, however, about 0.2 min shorter than those of the endogenously modified peptides. Furthermore, when observing the MS/MS fragmentation spectra of the peptides with MVK derivatized lysine, a characteristic neutral loss of 58 Da (C_3_H_6_O) was observed which could not be seen in the fragmentation spectra from the peptides with the endogenous modification. Figure 5A and B shows the MS/MS fragmentation spectra of peptide Q[+ 17]GLRGSLTK[+ 70]LK with the endogenous modification and with the artificial modification, respectively, and Fig. 5C shows the retention times of the same peptides. These data suggest that the chemical structure of the + 70 Da modification observed in the methionylated development sample of GM-CSF is not the reaction product of MVK.

Reacting the native recombinant GM-CSF with crotonaldehyde in vitro did not result in any detectable + 70 modification of the protein N-terminal or lysine residues (data not shown). The reaction did result in modification of histidine residues, which is not unexpected, since this amino acid is also a good nucleophile (Domingues et al. 2013).

## Discussion

### Investigation of the modification’s chemical structure and source

*E. coli* is one of the most employed expression systems for recombinant protein production. Especially for smaller proteins like GM-CSF that do not require specific PTMs for their activity and can be recovered in acceptable yields from inclusion bodies, *E. coli* offers the advantage of easy genetic manipulation and fast expression with a high yield at low cost (Wingfield 2015). Nevertheless, all recombinant protein expression is accompanied by the risk of introducing unspecific PTMs if the expression system is not monitored carefully, which may compromise the drugs stability and safety. Common PTMs observed during recombinant protein expression in *E. coli* are for example deamidation, proteolytic activity, incomplete N-terminal methionine cleavage, and disulfide scrambling, while less common attributes are internal starts in translation and oxidized protein products (Nagata et al. 1986; Wingfield 1987; Giglione et al. 2004; Nakamoto and Bardwell 2004). In the present study, an unexpected modification of + 70 Da was identified in process samples of GM-CSF expressed in *E. coli*. There are several protein modifications with different suggested chemical structures reported in the literature. By calculating the mass of the modification from peptide fragment ions and comparing this mass to a number of theoretical chemical structures, the elemental composition could be attributed to C_4_H_6_O (Fig. 3, Table 1). This limited the number of proposed candidates to the reaction products from butyryl-CoA, MVK, and crotonaldehyde. The modification’s ability to form derivatives with DNPH implicated the presence of a carbonyl moiety and proved that all three proposed sources were valid candidates (Fig. 4E and F). The list of possible candidates could then be further reduced, since the carbonyl carbon proved to be reducible by the mildly reducing agent borane pyridine complex (Fig. 4C and D). This implicated that the carbonyl carbon could not be involved in a strong bond, such as an amide bond, and butyryl-CoA could therefore be excluded as source to the modification. The last two candidates from our literature study for being the source to the identified + 70 Da modification were MVK and crotonaldehyde. Protein modification by MVK has been identified by von Stedingk et al. through Michael addition of the molecule to the N-terminal valine of human hemoglobin (von Stedingk et al. 2010). We set out to recreate this modification on GM-CSF, for comparison with the modification identified in our process samples, using commercially available MVK. We found that the group did readily react with lysine residues, but that the peptides had a slightly shifted retention time and a typical neutral loss of 58 Da which was not observed from the endogenous modification (Fig. 5). Ichihashi et al. identified crotonaldehyde as a potent chemical to react with nucleophilic amino acids such as lysine and histidine, proposedly through Michael addition (Ichihashi et al. 2001). Furthermore, Afonso et al. have studied the one carbon shorter alkenal, acrolein, and found that it was able to react with lysine residues of reduced lysozyme in a similar fashion (Afonso et al. 2018). When commercially available crotonaldehyde was reacted with GM-CSF in our current study, no reaction products of + 70 Da could be identified on the protein N-terminal or on lysine residues. There may be several explanations to this. One explanation could be that we did not manage to recreate the right environment for the reaction to appear. There are several parameters that may affect a successful reaction, such as pH, surrounding metabolites, and various enzymes. Another explanation could be that the wrong substrate was used for the reaction. In the nature crotonaldehyde exists as one out of two isomers, *cis* and *trans*. The commercially available crotonaldehyde is majorly in the *trans* conformation, so reactivity of the *cis* isoform could not be assayed. The + 70 Da modification may also have appeared from another source than crotonaldehyde. There are for example other substrates that in theory may result in the same reaction product as that from crotonaldehyde, one example being the metabolite crotonyl-CoA.

### Proposing crotonaldehyde as a possible source to the modification

Crotonaldehyde belongs to a group of aldehydes called 2-alkenals, which are known to be particularly susceptible to reaction with protein side chains. Due to their two electrophilic reaction centers, they are likely to be attacked by nucleophilic amino acids side chains, such as the primary amines of lysine and the protein N-terminal. Aldehydes have been identified as products when biological systems were exposed to oxidizing agents and to be causative agents to cytotoxic processes. For example, in the study by Ichihashi et al., crotonaldehyde modified proteins could be detected in renal tubules of rats that had been subjected to oxidative stress from Fe^3+^-NTA (Ichihashi et al. 2001). The formation of aldehydes in the presence of oxidative agents is suggested to proceed through lipid peroxidation, involving a number of free radical chain reaction mechanisms, and resulting in lipid hydroperoxides as the major initial reaction product (Esterbauer et al. 1991; Wu and Lin 1995). These can in turn decompose to several breakdown products from which aldehydes is among the more stable ones, compared to the free radicals. They can therefore diffuse within the cell and attack targets far from the original site and may thereby act as cytotoxic messengers. In the hope to identify any deviations in the expression conditions that may have caused the formation of crotonaldehyde, a number of parameters that were recorded in the reactor during expression of this specific batch were investigated. Interestingly, it was found that the oxygen levels in the incubator had been low for a longer period. Oxidative stress can be described as the state when reactive compounds is generated faster than the cell’s detoxification capacity, i.e., an imbalance in the redox balance within the cell (Georgiou 2002). During cell hypoxia, the ratio between NADH and NAD + usually increases due to insufficient O_2_ available to reduce NADH by the electron transport chain (Clanton 2007; Schulte et al. 2019). This accumulation of reducing equivalents makes electrons more available for reduction reactions leading to formation of reactive oxygen species (ROS) which may in turn initiate cascade reactions like lipid peroxidation. Hypoxia-induced lipid peroxidation has for example been observed in mouse embryonic fibroblasts and in blood from humans that had been exposed to periods of low oxygen supply (Joanny et al. 2001; Yajima et al. 2009). Another example where lipid peroxidation products have been identified is during ischemic reperfusion, when oxygen is allowed to return to the oxygen compromised cells (Cowled and Fitridge 2001). These observations support the theory that the + 70 Da modification has appeared as a result of an imbalance in the redox potential during protein expression caused by a time period of low oxygen pressure. This may in turn have led to the formation of lipid peroxidation products, such crotonaldehyde, that reacted with protein N-terminals and lysine residues to form the identified + 70 Da modification. However, no other adducts from lipid peroxidation byproducts, such as acrolein and hydroxynonenal, could be identified in the GM-CSF process sample.

### Protein carbonylation may affect therapeutic protein function and stability

Protein carbonylation has been related to aging as well as various diseases, such as Alzheimer’s disease, Parkinson’s disease, and atherosclerosis (Dalle-Donne et al. 2006). The introduction of carbonyls has been shown to cause protein dysfunction, either by blocking interaction sites or by changes in protein conformation. Improper folding may lead to protein aggregation followed by protein clearance. Carbonylation has also been shown to work as a marker for protein degradation in some cases. These facts highlight the importance to avoid introducing protein carbonylation products, such as the one characterized in this study, during therapeutic protein production by careful monitoring of all reaction parameters.

## Conclusion

In conclusion, we have identified and characterized a novel modification of + 70 Da located on lysine residues and on the protein N-terminal of rhGM-CSF. Based on current literature and on our experiments, we hypothesize that the chemical structure of the modification is the same as the reaction product of crotonaldehyde with a primary amine by Michael addition.

The source could, however, not be properly established, since the modification could not be recreated in vitro. Poorly controlled fermentation conditions are suspected to be related to the appearance of the modification.

### Supplementary Information

Below is the link to the electronic supplementary material.

Supplementary file1 (DOCX 170 kb)

## Figures and Tables

**Fig. 6:**
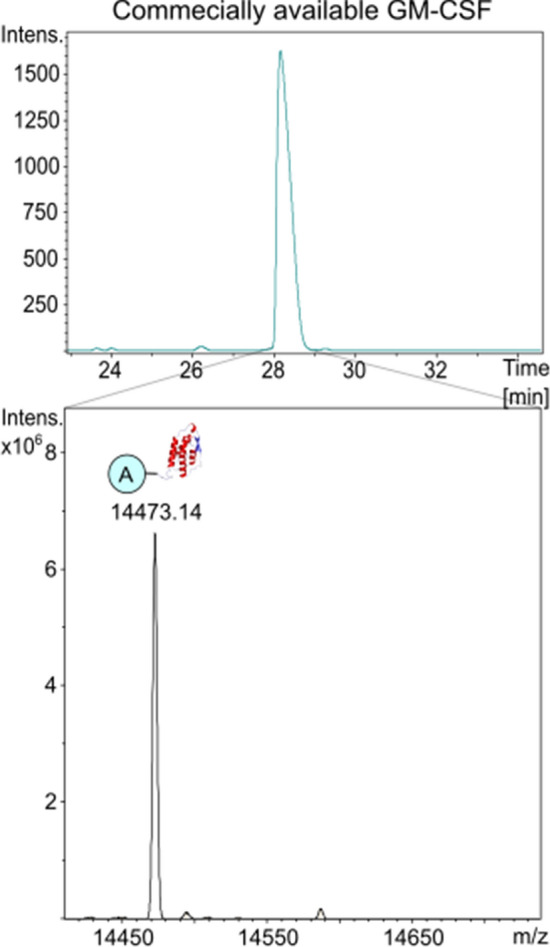
Commercially available rhGM-CSF was analyzed for comparison with the process samples as the intact protein by RP-LC coupled to UV (upper picture) and ESI–MS. The MS data from the main UV peak were deconvoluted to obtain the most abundant proteoforms (lower picture). The commercial GM-CSF contains mainly the native protein (with an N-terminal alanine residue)

**Fig. 7:**
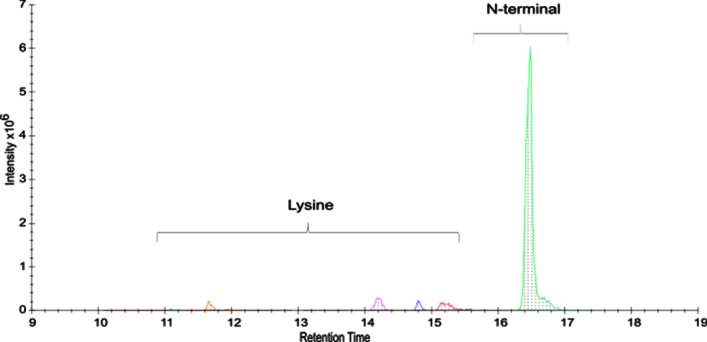
XICs of all peptides containing a + 70 Da modification from digestion with LysC + GluC and analyzed by LC-QTOF-MS. The peptides are grouped based on the location of the modification in the peptide

## Data Availability

The data that support the findings of this study are available upon request.

## Appendix

See Figs. 6, 7.

## Abbreviations

UHQ: Ultra-high quality
DNPH: 2,4-Dinitrophenylhydrazine
EVK: Ethyl vinyl ketone
MVK: Methyl vinyl ketone
GluC: Endoproteinase GluC
LysC: Endoproteinase LysC
DTT: 1,4-Dithiothreitol
TFA: Trifluoroacetic acid
FA: Formic acid
DMSO: Dimethyl sulfoxide
ACN: Acetonitrile
MWCO: Molecular weight cut-off
TEAB: Triethylammonium bicarbonate buffer
