# Supplementary material for: Characterization of a novel + 70 Da modification in rhGM-CSF expressed in E. coli using chemical assays in combination with mass spectrometry
Source: Amino Acids. 2021 Aug 28;54(4):601–13. doi: 10.1007/s00726-021-03004-9 (PMC9117350; doi:10.1007/s00726-021-03004-9)
Supplement: Supplementary file 1 — Supplementary file1 (DOCX 170 kb) [file 726_2021_3004_MOESM1_ESM.docx]

# Supplementary material

*Article title*: Characterization of a novel +70 Da modification in rhGM-CSF expressed in *E. coli* using chemical assays in combination with mass spectrometry

*Journal name*: Amino Acids - S.I.: PTM

*Authors*: Magdalena Widgren Sandberg^Aff1 & Aff2^, Jakob Bunkenborg^Aff2^, Stine Thyssen^Aff2^, Martin Villadsen ^Aff2^, Thomas Kofoed^Aff2^

*Aff1:* Universitätsklinikum Hamburg-Eppendorf (UKE), Hamburg, Germany

*Aff2*: Alphalyse A/S, Odense, Denmark

*Correspondence*: Magdalena Widgren Sandberg, [magdalena.w.sandberg@gmail.com](mailto:magdalena.w.sandberg@gmail.com)

## Derivatization of GM-CSF with pyruvic acid

### Background

In the same batch as the GM-CSF with a high degree of +70 Da modification was identified, Fig. 1 in the original article, proteoforms with an amino acid substitution from isoleucine to valine was also discovered. The amino acid substitution could be metabolically linked to the oxygen depletion in the expression medium leading to accumulation of valine through the intermediate pyruvate. Since pyruvate has been shown to form a modification of +70 Da with protein N-terminals and because of this close metabolic linkage an experiment was conducted to derivatize pyruvic acid with GM-CSF. The chromatographic and the fragmentation behavior of the pyruvic acid derivative should then be compared to that of the endogenous modification. This experiment was conducted despite the fact that the mass addition of a pyruvate moiety was found to diverge by around 500 ppm from the experimental value of the endogenous modification, Table 1 in the original article.

### Materials and Methods

*Derivatization with pyruvic acid:* A late process sample of GM-CSF which contained very low levels of +70 Da modified protein was incubated at 0.25 µg/µl with 100 mM pyruvic acid in 100 mM Tris, pH 9 for 48 h at 37 ֯C.

*Intact protein analysis by RP-LC-ESI-QTOF-MS:* 10 µg of the derivatized GM-CSF as well as the non-derivatized GM-CSF were analyzed intact as described in the original article under “Materials and Methods”, “Intact protein analysis by RP-LC-ESI-QTOF-MS”.

*Peptide mapping and data analysis:* The buffer of the derivatized sample was changed to 6 M urea in 50 mM sodium phosphate (NaP) buffer with pH 7 using Zeba Spin Desalting Columns, 7K MWCO, 0.5 mL (Thermo Fisher), according to the column protocol. The sample was then prepared and analyzed by RP-LC-MS/MS as described in the original article under “Materials and Methods” in “Protein digestion” followed by “Peptide SPE by HLB elution plate”, “Peptide mapping by RP-LC-ESI-TripleTOF-MS” and “Processing of peptide mapping data”.

### Results and Conclusion

A late process sample of GM-CSF which contained very low levels of +70 Da modified protein was incubated with 100 mM pyruvic acid in 100 mM Tris, pH 9 for 48 h. The derivatized sample was analyzed intact by RP-LC-MS together with the non-derivatized sample. Proteoforms of GM-CSF with additional masses of 70.1 Da and 87.9 Da could be identified in the derivatized sample but not in the non-derivatized sample, see Fig. S 1. The samples were therefore analyzed by peptide mapping and LC-MS/MS. The peak areas were quantified in Skyline. However, no peptides with a +70 Da adduct on the protein N-terminal or on lysine residues could be identified in higher abundance in the derivatized sample than in the non-derivatized sample. The conclusion was therefore that the +70 Da modification identified by intact LC-MS analysis did not form derivates with the same reactive groups as the endogenous modification or that it was not stable to LC-MS/MS analysis. In either case, the +70 Da modification identified in the early process samples of GM-CSF could therefore not be a pyruvate group.


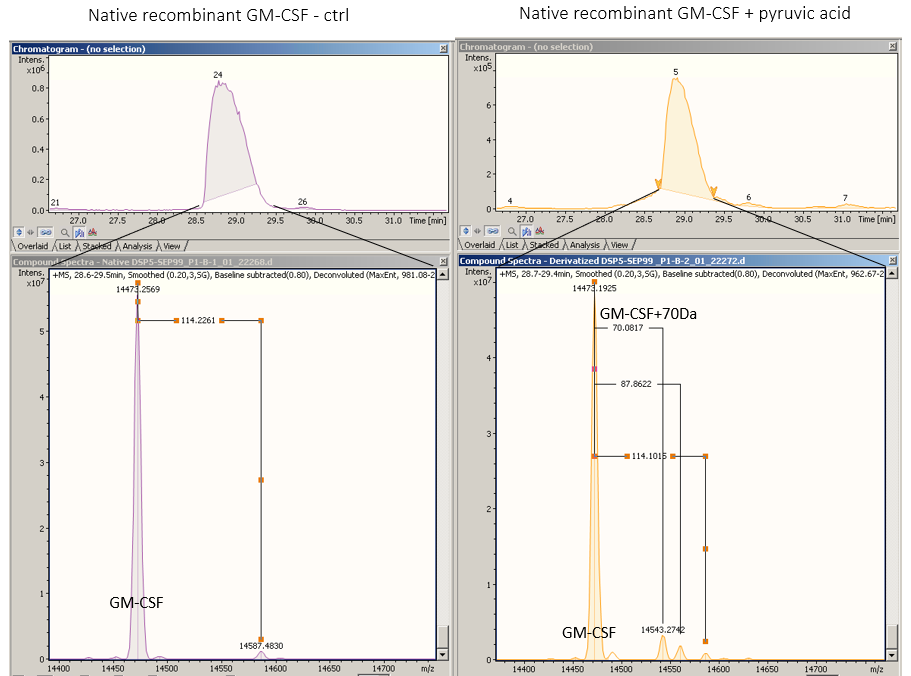


Fig. S 1: A late process sample with low levels of the +70 Da modification was derivatized with pyruvic acid and analyzed intact together with the non-derivatized GM-CSF by RP-LC coupled to UV (upper picture) and ESI-MS. The MS data from the main UV peak was deconvoluted to obtain the most abundant proteoforms (lower picture). **a** The non-derivatized GM-CSF, **b** The pyruvic acid derivatized GM-CSF.
